# 4-[(*tert*-Butyl­dimethyl­sil­yl)­oxy]-6-meth­oxy-7-methyl-5-(oxiran-2-ylmeth­yl)-2-benzofuran-3(1*H*)-one

**DOI:** 10.1107/S1600536811049026

**Published:** 2011-11-23

**Authors:** Magdalena Malachowska-Ugarte, Grzegorz Cholewinski, Jaroslaw Chojnacki, Krystyna Dzierzbicka

**Affiliations:** a Chemical Faculty, Gdansk University of Technology, Narutowicza 11/12, Gdansk PL-80233, Poland

## Abstract

The title compound, C_19_H_28_O_5_Si, was obtained in the reaction of 1,3-dihydro-4-[(*tert*-butyl­dimethyl­sil­yl)­oxy]-6-meth­oxy-7-methyl-3-oxo-5-(prop-2-en­yl)isobenzofuran with *meta*-chloro­perbenzoic acid. This reaction is one of the stages of the total synthesis of mycophenolic acid, which we attempted to modify. The title compound forms crystals with only weak inter­molecular inter­actions. The strongest stacking inter­action is found between the benzene and furan rings of inversion-related mol­ecules with a distance of 3.8773 (13) Å between the ring centroids.

## Related literature

For structures of related oxiranes, see: Langer & Becker (1993 ▶); Berthalon *et al.* (1999 ▶). For the preparation of the title compound, see: Patterson (1995 ▶); Plé *et al.* (1997 ▶). For the properties of epoxides, see: Padwa & Murphree (2006 ▶). For a description of the Cambridge Structural Database, see Allen (2002 ▶).
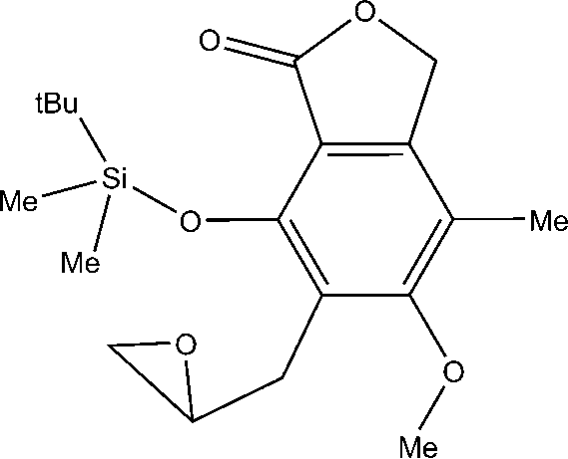


## Experimental

### 

#### Crystal data

C_19_H_28_O_5_Si
*M* *_r_* = 364.5
Monoclinic, 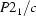

*a* = 7.5682 (3) Å
*b* = 12.2488 (4) Å
*c* = 20.6905 (8) Å
β = 93.990 (4)°
*V* = 1913.39 (12) Å^3^
*Z* = 4
Mo *K*α radiation
μ = 0.15 mm^−1^
*T* = 120 K
0.55 × 0.44 × 0.35 mm

#### Data collection

Agilent Xcalibur diffractometer
Absorption correction: analytical (Clark & Reid, 1995 ▶) *T*_min_ = 0.938, *T*_max_ = 0.954
6727 measured reflections
3430 independent reflections
2858 reflections with *I* > 2σ(*I*)
*R*_int_ = 0.017

#### Refinement

*R*[*F* ^2^ > 2σ(*F* ^2^)] = 0.045
*wR*(*F* ^2^) = 0.123
*S* = 1.09
3430 reflections
243 parameters
2 restraints
H-atom parameters constrained
Δρ_max_ = 0.42 e Å^−3^
Δρ_min_ = −0.18 e Å^−3^

### 

Data collection: *CrysAlis PRO* (Agilent, 2010 ▶); cell refinement: *CrysAlis PRO*; data reduction: *CrysAlis PRO*; program(s) used to solve structure: *SUPERFLIP* (Palatinus & Chapuis, 2007 ▶); program(s) used to refine structure: *SHELXL97* (Sheldrick, 2008 ▶); molecular graphics: *ORTEP-3* (Farrugia, 1997 ▶); software used to prepare material for publication: *publCIF* (Westrip, 2010 ▶), *PLATON* (Spek, 2009 ▶), *WinGX* (Farrugia, 1999 ▶) and *Mercury* (Macrae *et al.*, 2006 ▶).

## Supplementary Material

Crystal structure: contains datablock(s) global, I. DOI: 10.1107/S1600536811049026/fy2029sup1.cif

Structure factors: contains datablock(s) I. DOI: 10.1107/S1600536811049026/fy2029Isup2.hkl

Supplementary material file. DOI: 10.1107/S1600536811049026/fy2029Isup3.cml

Additional supplementary materials:  crystallographic information; 3D view; checkCIF report

## Figures and Tables

**Fig. 1.**
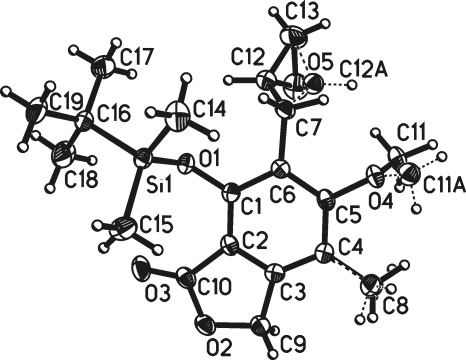
Molecular structure of C19H28O5Si, showing the atomic labeling scheme. Displacement ellipsoids are drawn at the 50% probability level.

## supplementary crystallographic information

### Comment

Presented research is an attempt to modify the known multi-stage total synthesis of mycophenolic acid (Patterson, 1995) by making use of epoxides as intermediates. The synthesis of the desired epoxide was successful and its X-ray structure has been determined.

In the title compound, bond length C12—C13 is *ca* 0.01 Å longer than C12—O5 or C13—O5, which is a trend noted for other oxirans. The valence angle at O5 is close to the average of 60.5 (9)° calculated for 17 structures containing benzyloxirane fragment, according to our CSD search (Allen *et al.*, 2002). The most closely related compounds, which contain the 1-phenyl-2,3-epoxypropane fragment are *rac*-3-(9-anthryl)-1,2-epoxypropane (Langer *et al.*, 1993) and a modified calix[4]arene (Berthalon *et al.*, 1999).

The crystal lattice is composed of discrete molecules with no strong specific intermolecular interactions. The strongest stacking interaction is found between the benzene and furan rings of molecules related by the inversion at (1/2, 1/2, 1) with a distance of 3.8773 (13) Å between the ring centroids.

The *ORTEP* view of the title epoxide is given in Fig. 1. Although the oxirane ring is not connected with the aromatic ring directly, the shortest intramolecular contact between the ring and adjacent methoxy substituent is rather short 2.560 (3) Å for O5···H11A. Epoxides are known to be reactive compounds (Padwa *et al.*, 2006), but the investigated oxirane is relatively stable and can be purified on silica gel and stored for several months. We suppose the reactivity of the epoxide ring is decreased by the steric hindrance from the proximate methoxyl and *t*-BuMe_2_Si substituents.

### Experimental

The starting material 1,3-dihydro-4-[(*tert*-butyldimethylsilyl)oxy]-6-methoxy-7-methyl-3-oxo-5-(prop-2-enyl)isobenzofuran was obtained according to the chemical literature (Patterson, 1995).

Preparation of 1,3-dihydro-4-[(*tert*-butyldimethylsilyl)oxy]-6-methoxy-7-methyl-3-oxo-5-(2,3-epoxypropanyl)isobenzofuran, was carried out based on the procedure reported in the chemical literature (Plé *et al.*, 1997). In the cited work geranyl acetate was oxidized to 6,7-epoxygeranyl acetate with *meta*-chloroperoxybenzoic acid (*m*-CPBA). We applied lower temperature for addition of *m*-CPBA (–70 °C, instead of – 30 °C), and then the reaction was carried out at room temperature (instead of 0 °C).

The solution of 1,3-dihydro-4-[(*tert*-butyldimethylsilyl)oxy]-6-methoxy-7-methyl-3-oxo-5-(prop-2-enyl)isobenzofuran (6.5 mmol), freshly melted sodium acetate (6.5 mmol) in anhydrous methyl chloride (20 ml) was cooled to –70 °C. Subsequently, *m*-CPBA (13 mmol) (*meta*-chloroperbenzoic acid) was added portionwise and the reaction mixture was stirred at room temperature for 5 h. Then it was washed with diluted NaHCO_3_, and the aqueous layer was extracted with methylene chloride. Next, the combined organic layers were washed with cooled 1*M* NaOH, dried over Na_2_SO_4_ and filtered and evaporated under vacuum. The crude product was purified with column chromatography (petroleum ether – ethyl acetate 10:1) to give 3-dihydro-4-[(*tert*-butyldimethylsilyl)oxy]-6-methoxy-7-methyl-3-oxo-5-(2,3-epoxypropanyl)isobenzofuran in 80% yield.

Single crystals were obtained by vapour diffusion of petroleum ether into a solution of about 30 mg product in 1 mL dichloromethane over 3-4 days (m.p. 96–98 °C).

### Refinement

All hydrogen atoms were refined in isotropic approximation with *U* values fixed to be 1.5 times *U_eq_* of C atoms for CH_3_ or 1.2 times *U_eq_* for CH_2_ and CH groups. C12 oxiran atom was found disordered over two positions (with 0.839 (6)/0.161 (6) probablilities). The same splitting ratio was applied to the disorder of the neighbouring O4—C13 methoxy group and the second neighbour C8 methyl group to avoid short contacts within the molecule.

### Crystal data

**Table:**

C_19_H_28_O_5_Si	*F*(000) = 784
*M**_r_* = 364.5	*D*_x_ = 1.265 Mg m^−^^3^
Monoclinic, *P*2_1_/*c*	Melting point: 369(2) K
Hall symbol: -P 2ybc	Mo *K*α radiation, λ = 0.71073 Å
*a* = 7.5682 (3) Å	Cell parameters from 4883 reflections
*b* = 12.2488 (4) Å	θ = 2.6–28.8°
*c* = 20.6905 (8) Å	µ = 0.15 mm^−^^1^
β = 93.990 (4)°	*T* = 120 K
*V* = 1913.39 (12) Å^3^	Block, colourless
*Z* = 4	0.55 × 0.44 × 0.35 mm

### Data collection

**Table:**

Agilent Xcalibur diffractometer	3430 independent reflections
graphite	2858 reflections with *I* > 2σ(*I*)
Detector resolution: 8.1883 pixels mm^-1^	*R*_int_ = 0.017
ω scans	θ_max_ = 25.2°, θ_min_ = 2.6°
Absorption correction: analytical (Clark & Reid, 1995)	*h* = −9→7
*T*_min_ = 0.938, *T*_max_ = 0.954	*k* = −13→14
6727 measured reflections	*l* = −23→24

### Refinement

**Table:**

Refinement on *F*^2^	Primary atom site location: structure-invariant direct methods
Least-squares matrix: full	Secondary atom site location: difference Fourier map
*R*[*F*^2^ > 2σ(*F*^2^)] = 0.045	Hydrogen site location: inferred from neighbouring sites
*wR*(*F*^2^) = 0.123	H-atom parameters constrained
*S* = 1.09	*w* = 1/[σ^2^(*F*_o_^2^) + (0.075*P*)^2^ + 0.4245*P*] where *P* = (*F*_o_^2^ + 2*F*_c_^2^)/3
3430 reflections	(Δ/σ)_max_ = 0.013
243 parameters	Δρ_max_ = 0.42 e Å^−^^3^
2 restraints	Δρ_min_ = −0.18 e Å^−^^3^

### Special details

**Table:**

Experimental. ^1^H NMR (CDCl_3,_δ): 0.25 (s, 3H), 0.26 (s, 3H), 1.04 (s, 9H), 2.18 (s, 3H), 2.55 (dd, J = 5.1, 2.7, 1H), 2.70 (dd, J = 4.4, 4.4, 1H), 2.86 (dd, J = 13.7, 5.9, 1H), 3.10 (dd, J = 13.7, 4.9, 1H), 3.18 – 3.19 (m, 1H), 3.81 (s, 3H), 5.09 (s, 2H).^13^C NMR (CDCl_3,_δ): -3.35, -3.23, 1.25, 11.74, 18.98, 26.26, 27.92, 47.41, 51.29, 61.30, 67.93, 111.99, 118.31, 123.41, 147.18, 152.44, 163.72, 169.25.MS: C_19_H_28_O_5_Si *M*/*z* = 364.3 (calculated 364.2)NMR spectra were recorded with Varian Unity Plus 500 MHz. Coupling constants are given in Hz. Mass spectrum was recorded with MALDI-TOF spectrometer BRUKER BIFLEX III (DHB matrix).Column chromatography was carried out on silica gel Merck 60 (0.063–0.2 mm). The reactions were followed with TLC technique on plates Merck 60 F_254_. All solvents were purified according to standard methods.
Geometry. All s.u.'s (except the s.u. in the dihedral angle between two l.s. planes) are estimated using the full covariance matrix. The cell s.u.'s are taken into account individually in the estimation of s.u.'s in distances, angles and torsion angles; correlations between s.u.'s in cell parameters are only used when they are defined by crystal symmetry. An approximate (isotropic) treatment of cell s.u.'s is used for estimating s.u.'s involving l.s. planes.
Refinement. Refinement of *F*^2^ against ALL reflections. The weighted *R*-factor *wR* and goodness of fit *S* are based on *F*^2^, conventional *R*-factors *R* are based on *F*, with *F* set to zero for negative *F*^2^. The threshold expression of *F*^2^ > 2σ(*F*^2^) is used only for calculating *R*-factors(gt) *etc*. and is not relevant to the choice of reflections for refinement. *R*-factors based on *F*^2^ are statistically about twice as large as those based on *F*, and *R*- factors based on ALL data will be even larger.

### Fractional atomic coordinates and isotropic or equivalent isotropic displacement parameters (Å^2^)

**Table:**

	*x*	*y*	*z*	*U*_iso_*/*U*_eq_	Occ. (<1)
Si1	0.75384 (6)	0.75814 (4)	0.80053 (2)	0.02184 (17)	
O1	0.74792 (15)	0.71310 (10)	0.87721 (6)	0.0229 (3)	
O2	0.42666 (18)	0.41649 (10)	0.87895 (6)	0.0306 (3)	
O3	0.67752 (19)	0.46983 (11)	0.83748 (7)	0.0370 (4)	
O4	0.31864 (16)	0.82981 (10)	1.02105 (6)	0.0268 (3)	
C1	0.6018 (2)	0.69206 (14)	0.91006 (8)	0.0208 (4)	
C2	0.5145 (2)	0.59172 (14)	0.90497 (8)	0.0220 (4)	
C3	0.3654 (2)	0.57202 (14)	0.93807 (8)	0.0228 (4)	
C4	0.2933 (2)	0.64859 (14)	0.97795 (8)	0.0233 (4)	
C5	0.3860 (2)	0.74744 (14)	0.98426 (8)	0.0226 (4)	
C6	0.5399 (2)	0.77006 (13)	0.95246 (8)	0.0210 (4)	
C7	0.6395 (2)	0.87568 (14)	0.96487 (9)	0.0265 (4)	
H7A	0.6557	0.912	0.9229	0.032*	
H7B	0.5679	0.9247	0.9907	0.032*	
C8	0.1239 (18)	0.6273 (9)	1.0104 (6)	0.0328 (8)	0.839 (5)
H8A	0.0916	0.6925	1.0343	0.049*	0.839 (5)
H8B	0.1415	0.5658	1.0405	0.049*	0.839 (5)
H8C	0.0289	0.6097	0.9774	0.049*	0.839 (5)
C8A	0.128 (10)	0.628 (5)	1.007 (3)	0.0328 (8)	0.161 (5)
H8D	0.0882	0.5537	0.9972	0.049*	0.161 (5)
H8E	0.0385	0.6803	0.9905	0.049*	0.161 (5)
H8F	0.1467	0.6366	1.0545	0.049*	0.161 (5)
C9	0.3019 (3)	0.45817 (14)	0.92251 (9)	0.0276 (4)	
H9A	0.3025	0.4131	0.9623	0.033*	
H9B	0.1805	0.4592	0.9014	0.033*	
C10	0.5564 (3)	0.49177 (15)	0.86926 (9)	0.0272 (4)	
C11	0.3706 (5)	0.8213 (3)	1.08945 (13)	0.0311 (7)	0.839 (5)
H11A	0.4995	0.8286	1.0962	0.047*	0.839 (5)
H11B	0.3342	0.7501	1.1056	0.047*	0.839 (5)
H11C	0.3133	0.8794	1.1129	0.047*	0.839 (5)
C11A	0.304 (2)	0.8127 (17)	1.0872 (10)	0.035 (5)*	0.161 (5)
H11D	0.2542	0.8781	1.1064	0.052*	0.161 (5)
H11E	0.4211	0.7976	1.1083	0.052*	0.161 (5)
H11F	0.2254	0.7504	1.0933	0.052*	0.161 (5)
O5	0.8139 (2)	0.82094 (13)	1.06596 (7)	0.0447 (4)	
C12	0.8155 (3)	0.85929 (19)	0.99979 (11)	0.0303 (7)	0.839 (5)
H12	0.9079	0.826	0.9737	0.036*	0.839 (5)
C12A	0.7066 (14)	0.8869 (9)	1.0375 (5)	0.029 (3)*	0.161 (5)
H12A	0.6155	0.916	1.0657	0.034*	0.161 (5)
C13	0.8822 (3)	0.9282 (2)	1.05370 (11)	0.0482 (6)	
H13A	1.0118	0.9393	1.0599	0.058*	
H13B	0.8097	0.9918	1.0649	0.058*	
C14	0.6788 (3)	0.90278 (17)	0.79575 (10)	0.0375 (5)	
H14A	0.7587	0.948	0.8238	0.056*	
H14B	0.5583	0.908	0.81	0.056*	
H14C	0.68	0.9285	0.7509	0.056*	
C15	0.6074 (3)	0.67721 (18)	0.74361 (9)	0.0366 (5)	
H15A	0.4865	0.6782	0.7578	0.055*	
H15B	0.6501	0.6017	0.7428	0.055*	
H15C	0.6078	0.7088	0.7001	0.055*	
C16	0.9937 (2)	0.74546 (15)	0.78426 (9)	0.0259 (4)	
C17	1.1085 (3)	0.81497 (18)	0.83260 (10)	0.0382 (5)	
H17A	1.0757	0.892	0.8273	0.057*	
H17B	1.2336	0.8057	0.8245	0.057*	
H17C	1.0894	0.7916	0.8769	0.057*	
C18	1.0503 (3)	0.62553 (18)	0.79128 (13)	0.0440 (6)	
H18A	1.1764	0.6189	0.7841	0.066*	
H18B	0.9809	0.5812	0.7593	0.066*	
H18C	1.0296	0.5998	0.835	0.066*	
C19	1.0203 (3)	0.7855 (2)	0.71551 (10)	0.0414 (5)	
H19A	0.9473	0.7417	0.6843	0.062*	
H19B	1.1453	0.778	0.7067	0.062*	
H19C	0.9853	0.8623	0.7115	0.062*	

### Atomic displacement parameters (Å^2^)

**Table:**

	*U*^11^	*U*^22^	*U*^33^	*U*^12^	*U*^13^	*U*^23^
Si1	0.0212 (3)	0.0250 (3)	0.0195 (3)	0.00013 (19)	0.00251 (19)	0.00215 (19)
O1	0.0221 (6)	0.0270 (6)	0.0202 (6)	0.0007 (5)	0.0055 (5)	0.0011 (5)
O2	0.0433 (8)	0.0202 (6)	0.0289 (7)	−0.0026 (6)	0.0072 (6)	−0.0032 (5)
O3	0.0470 (9)	0.0279 (7)	0.0381 (8)	0.0057 (6)	0.0169 (7)	−0.0044 (6)
O4	0.0317 (7)	0.0235 (6)	0.0259 (7)	0.0063 (5)	0.0061 (5)	−0.0033 (5)
C1	0.0226 (9)	0.0226 (9)	0.0173 (8)	0.0013 (7)	0.0015 (7)	0.0030 (7)
C2	0.0269 (9)	0.0220 (9)	0.0171 (8)	0.0043 (7)	0.0010 (7)	0.0012 (7)
C3	0.0263 (9)	0.0221 (9)	0.0200 (9)	−0.0014 (7)	−0.0001 (7)	0.0031 (7)
C4	0.0234 (9)	0.0241 (9)	0.0226 (9)	0.0022 (7)	0.0025 (7)	0.0029 (7)
C5	0.0269 (9)	0.0208 (9)	0.0200 (9)	0.0062 (7)	0.0022 (7)	0.0006 (7)
C6	0.0241 (9)	0.0195 (8)	0.0193 (9)	0.0009 (7)	0.0009 (7)	0.0015 (7)
C7	0.0348 (10)	0.0197 (9)	0.0256 (10)	−0.0026 (7)	0.0063 (8)	−0.0024 (7)
C8	0.0308 (12)	0.0301 (11)	0.039 (2)	−0.0030 (9)	0.0112 (12)	−0.0024 (9)
C8A	0.0308 (12)	0.0301 (11)	0.039 (2)	−0.0030 (9)	0.0112 (12)	−0.0024 (9)
C9	0.0346 (10)	0.0236 (9)	0.0253 (10)	−0.0022 (8)	0.0056 (8)	−0.0001 (8)
C10	0.0377 (11)	0.0212 (9)	0.0231 (9)	0.0015 (8)	0.0037 (8)	0.0001 (7)
C11	0.0414 (19)	0.0319 (15)	0.0207 (13)	0.0061 (15)	0.0065 (14)	−0.0039 (10)
O5	0.0453 (9)	0.0544 (10)	0.0332 (8)	0.0020 (7)	−0.0055 (7)	0.0002 (7)
C12	0.0284 (13)	0.0305 (13)	0.0321 (13)	−0.0021 (10)	0.0025 (10)	−0.0054 (10)
C13	0.0406 (12)	0.0521 (14)	0.0503 (14)	−0.0084 (11)	−0.0072 (11)	−0.0174 (11)
C14	0.0444 (12)	0.0354 (11)	0.0334 (11)	0.0099 (9)	0.0070 (9)	0.0085 (9)
C15	0.0345 (11)	0.0476 (12)	0.0269 (10)	−0.0115 (9)	−0.0037 (8)	0.0061 (9)
C16	0.0228 (9)	0.0308 (10)	0.0247 (10)	−0.0026 (7)	0.0054 (7)	−0.0025 (8)
C17	0.0262 (10)	0.0504 (13)	0.0378 (12)	−0.0071 (9)	0.0000 (9)	−0.0083 (10)
C18	0.0325 (11)	0.0380 (12)	0.0630 (15)	0.0085 (9)	0.0140 (10)	−0.0017 (11)
C19	0.0405 (12)	0.0563 (13)	0.0291 (11)	−0.0124 (10)	0.0147 (9)	−0.0018 (10)

### Geometric parameters (Å, °)

**Table:**

Si1—O1	1.6832 (12)	C11—H11A	0.98
Si1—C15	1.849 (2)	C11—H11B	0.98
Si1—C14	1.861 (2)	C11—H11C	0.98
Si1—C16	1.8756 (19)	C11A—H11D	0.98
O1—C1	1.363 (2)	C11A—H11E	0.98
O2—C10	1.372 (2)	C11A—H11F	0.98
O2—C9	1.443 (2)	O5—C12A	1.262 (11)
O3—C10	1.196 (2)	O5—C13	1.440 (3)
O4—C5	1.382 (2)	O5—C12	1.448 (3)
O4—C11A	1.40 (2)	C12—C13	1.460 (3)
O4—C11	1.446 (3)	C12—H12	1
C1—C2	1.396 (2)	C12A—C13	1.439 (10)
C1—C6	1.400 (2)	C12A—H12A	1
C2—C3	1.381 (2)	C13—H13A	0.99
C2—C10	1.476 (2)	C13—H13B	0.99
C3—C4	1.386 (2)	C14—H14A	0.98
C3—C9	1.502 (2)	C14—H14B	0.98
C4—C5	1.401 (3)	C14—H14C	0.98
C4—C8A	1.45 (8)	C15—H15A	0.98
C4—C8	1.510 (15)	C15—H15B	0.98
C5—C6	1.405 (2)	C15—H15C	0.98
C6—C7	1.510 (2)	C16—C19	1.531 (3)
C7—C12	1.484 (3)	C16—C18	1.534 (3)
C7—C12A	1.559 (10)	C16—C17	1.536 (3)
C7—H7A	0.99	C17—H17A	0.98
C7—H7B	0.99	C17—H17B	0.98
C8—H8A	0.98	C17—H17C	0.98
C8—H8B	0.98	C18—H18A	0.98
C8—H8C	0.98	C18—H18B	0.98
C8A—H8D	0.98	C18—H18C	0.98
C8A—H8E	0.98	C19—H19A	0.98
C8A—H8F	0.98	C19—H19B	0.98
C9—H9A	0.99	C19—H19C	0.98
C9—H9B	0.99		
			
O1—Si1—C15	111.75 (8)	O4—C11A—H11E	109.5
O1—Si1—C14	109.52 (8)	H11D—C11A—H11E	109.5
C15—Si1—C14	108.02 (10)	O4—C11A—H11F	109.5
O1—Si1—C16	103.39 (7)	H11D—C11A—H11F	109.5
C15—Si1—C16	112.72 (9)	H11E—C11A—H11F	109.5
C14—Si1—C16	111.40 (9)	C12A—O5—C13	63.9 (5)
C1—O1—Si1	127.45 (11)	C12A—O5—C12	53.0 (5)
C10—O2—C9	111.07 (13)	C13—O5—C12	60.73 (14)
C5—O4—C11A	119.2 (9)	O5—C12—C13	59.37 (15)
C5—O4—C11	113.62 (16)	O5—C12—C7	115.98 (18)
O1—C1—C2	121.69 (15)	C13—C12—C7	123.0 (2)
O1—C1—C6	120.15 (15)	O5—C12—H12	115.5
C2—C1—C6	118.10 (16)	C13—C12—H12	115.5
C3—C2—C1	121.01 (16)	C7—C12—H12	115.5
C3—C2—C10	108.34 (15)	O5—C12A—C13	64.1 (5)
C1—C2—C10	130.62 (16)	O5—C12A—C7	123.3 (8)
C2—C3—C4	123.09 (16)	C13—C12A—C7	119.3 (7)
C2—C3—C9	108.42 (15)	O5—C12A—H12A	113.8
C4—C3—C9	128.48 (16)	C13—C12A—H12A	113.8
C3—C4—C5	115.14 (16)	C7—C12A—H12A	113.8
C3—C4—C8A	121 (2)	O5—C12A—H13B	93.9
C5—C4—C8A	123 (2)	C7—C12A—H13B	122.4
C3—C4—C8	121.9 (5)	H12A—C12A—H13B	81.1
C5—C4—C8	122.9 (5)	C12A—C13—O5	52.0 (5)
O4—C5—C4	118.72 (15)	C12A—C13—C12	49.8 (5)
O4—C5—C6	117.61 (15)	O5—C13—C12	59.90 (14)
C4—C5—C6	123.58 (16)	O5—C13—H13A	117.8
C1—C6—C5	118.91 (15)	C12—C13—H13A	117.8
C1—C6—C7	120.43 (15)	C12A—C13—H13B	79.2
C5—C6—C7	120.65 (15)	O5—C13—H13B	117.7
C12—C7—C6	112.79 (16)	C12—C13—H13B	117.8
C12—C7—C12A	47.3 (4)	H13A—C13—H13B	114.9
C6—C7—C12A	111.4 (4)	Si1—C14—H14A	109.5
C12—C7—H7A	109	Si1—C14—H14B	109.5
C6—C7—H7A	109	H14A—C14—H14B	109.5
C12A—C7—H7A	138.9	Si1—C14—H14C	109.5
C12—C7—H7B	109	H14A—C14—H14C	109.5
C6—C7—H7B	109	H14B—C14—H14C	109.5
C12A—C7—H7B	65.2	Si1—C15—H15A	109.5
H7A—C7—H7B	107.8	Si1—C15—H15B	109.5
C4—C8—H8A	109.5	H15A—C15—H15B	109.5
C4—C8—H8B	109.5	Si1—C15—H15C	109.5
H8A—C8—H8B	109.5	H15A—C15—H15C	109.5
C4—C8—H8C	109.5	H15B—C15—H15C	109.5
H8A—C8—H8C	109.5	C19—C16—C18	109.91 (17)
H8B—C8—H8C	109.5	C19—C16—C17	108.83 (16)
C4—C8A—H8D	109.5	C18—C16—C17	109.13 (17)
C4—C8A—H8E	109.5	C19—C16—Si1	109.37 (13)
H8D—C8A—H8E	109.5	C18—C16—Si1	109.24 (13)
C4—C8A—H8F	109.5	C17—C16—Si1	110.36 (13)
H8D—C8A—H8F	109.5	C16—C17—H17A	109.5
H8E—C8A—H8F	109.5	C16—C17—H17B	109.5
O2—C9—C3	104.40 (14)	H17A—C17—H17B	109.5
O2—C9—H9A	110.9	C16—C17—H17C	109.5
C3—C9—H9A	110.9	H17A—C17—H17C	109.5
O2—C9—H9B	110.9	H17B—C17—H17C	109.5
C3—C9—H9B	110.9	C16—C18—H18A	109.5
H9A—C9—H9B	108.9	C16—C18—H18B	109.5
O3—C10—O2	120.90 (16)	H18A—C18—H18B	109.5
O3—C10—C2	131.42 (18)	C16—C18—H18C	109.5
O2—C10—C2	107.67 (15)	H18A—C18—H18C	109.5
O4—C11—H11A	109.5	H18B—C18—H18C	109.5
O4—C11—H11B	109.5	C16—C19—H19A	109.5
H11A—C11—H11B	109.5	C16—C19—H19B	109.5
O4—C11—H11C	109.5	H19A—C19—H19B	109.5
H11A—C11—H11C	109.5	C16—C19—H19C	109.5
H11B—C11—H11C	109.5	H19A—C19—H19C	109.5
O4—C11A—H11D	109.5	H19B—C19—H19C	109.5
			
C15—Si1—O1—C1	−49.33 (16)	C10—O2—C9—C3	1.70 (19)
C14—Si1—O1—C1	70.33 (16)	C2—C3—C9—O2	0.31 (19)
C16—Si1—O1—C1	−170.83 (14)	C4—C3—C9—O2	−179.97 (16)
Si1—O1—C1—C2	85.05 (19)	C9—O2—C10—O3	175.75 (17)
Si1—O1—C1—C6	−97.84 (17)	C9—O2—C10—C2	−2.94 (19)
O1—C1—C2—C3	−179.33 (15)	C3—C2—C10—O3	−175.4 (2)
C6—C1—C2—C3	3.5 (2)	C1—C2—C10—O3	2.7 (3)
O1—C1—C2—C10	2.8 (3)	C3—C2—C10—O2	3.1 (2)
C6—C1—C2—C10	−174.42 (17)	C1—C2—C10—O2	−178.78 (16)
C1—C2—C3—C4	−0.1 (3)	C12A—O5—C12—C13	78.0 (6)
C10—C2—C3—C4	178.22 (16)	C12A—O5—C12—C7	−36.6 (6)
C1—C2—C3—C9	179.64 (15)	C13—O5—C12—C7	−114.6 (2)
C10—C2—C3—C9	−2.03 (19)	C6—C7—C12—O5	−67.0 (2)
C2—C3—C4—C5	−2.0 (3)	C12A—C7—C12—O5	31.7 (6)
C9—C3—C4—C5	178.31 (17)	C6—C7—C12—C13	−136.0 (2)
C2—C3—C4—C8A	175 (3)	C12A—C7—C12—C13	−37.3 (6)
C9—C3—C4—C8A	−5(3)	C12—O5—C12A—C13	−71.7 (4)
C2—C3—C4—C8	176.3 (5)	C13—O5—C12A—C7	109.4 (9)
C9—C3—C4—C8	−3.4 (6)	C12—O5—C12A—C7	37.6 (6)
C11A—O4—C5—C4	63.1 (9)	C12—C7—C12A—O5	−40.4 (6)
C11—O4—C5—C4	85.5 (2)	C6—C7—C12A—O5	61.4 (9)
C11A—O4—C5—C6	−120.1 (9)	C12—C7—C12A—C13	36.2 (5)
C11—O4—C5—C6	−97.7 (2)	C6—C7—C12A—C13	138.0 (7)
C3—C4—C5—O4	177.35 (15)	C7—C12A—C13—O5	−115.3 (10)
C8A—C4—C5—O4	1(3)	O5—C12A—C13—C12	80.0 (5)
C8—C4—C5—O4	−0.9 (6)	C7—C12A—C13—C12	−35.3 (5)
C3—C4—C5—C6	0.8 (3)	C12—O5—C13—C12A	60.4 (6)
C8A—C4—C5—C6	−176 (3)	C12A—O5—C13—C12	−60.4 (6)
C8—C4—C5—C6	−177.5 (6)	O5—C12—C13—C12A	−63.8 (6)
O1—C1—C6—C5	178.18 (14)	C7—C12—C13—C12A	39.1 (6)
C2—C1—C6—C5	−4.6 (2)	C7—C12—C13—O5	102.8 (2)
O1—C1—C6—C7	−3.2 (2)	O1—Si1—C16—C19	−179.28 (13)
C2—C1—C6—C7	174.04 (15)	C15—Si1—C16—C19	59.87 (16)
O4—C5—C6—C1	−174.07 (15)	C14—Si1—C16—C19	−61.75 (16)
C4—C5—C6—C1	2.6 (3)	O1—Si1—C16—C18	60.38 (15)
O4—C5—C6—C7	7.3 (2)	C15—Si1—C16—C18	−60.46 (17)
C4—C5—C6—C7	−176.07 (16)	C14—Si1—C16—C18	177.91 (14)
C1—C6—C7—C12	−66.9 (2)	O1—Si1—C16—C17	−59.59 (15)
C5—C6—C7—C12	111.68 (19)	C15—Si1—C16—C17	179.56 (14)
C1—C6—C7—C12A	−118.2 (5)	C14—Si1—C16—C17	57.94 (16)
C5—C6—C7—C12A	60.4 (5)		
